# Cognitive Test Performance in CLN3 Disease Is Associated With the CLN3 Staging System (CLN3SS)

**DOI:** 10.1002/jmd2.70136

**Published:** 2026-11-05

**Authors:** Samuel D. Moran, Erika F. Augustine, Jonathan W. Mink, Marianna Pereira‐Freitas, Naomi S. Taggart, Jennifer Vermilion, Amy E. Vierhile, Heather R. Adams

**Affiliations:** ^1^ University of Rochester School of Medicine and Dentistry Rochester New York USA; ^2^ Kennedy Krieger Institute Baltimore Maryland USA; ^3^ Department of Neurology University of Rochester Medicine Rochester New York USA; ^4^ Independent Consultant Pittsford New York USA

**Keywords:** Batten disease, CLN3 disease, CLN3 staging system, cognition, disease staging, juvenile neuronal ceroid lipofuscinosis

## Abstract

CLN3 disease is an inherited neurodegenerative disease, typically with childhood onset, and characterized by vision loss, seizures, cognitive decline, and difficulties. The CLN3 Staging System (CLN3SS) characterizes disease progression. Our aim was to assess differences in cognitive test scores in relation to CLN3SS among individuals with CLN3 disease. We evaluated the relationship between cognitive test performance and the CLN3SS in individuals with genetically confirmed CLN3 disease. Participants completed tasks of verbal reasoning, vocabulary knowledge, attention, fund of information, and ability to recite the alphabet. One‐way ANOVA testing assessed differences in mean cognitive test score among CLN3SS score groups, and chi‐square testing was used to compare the proportion in each CLN3SS group that could recite the alphabet. Data were evaluated from a sample of 85 individuals with a total 245 CLN3SS assessments conducted within 6 months of their cognitive testing, A significant decrease in test scores was found between CLN3SS Stages 1 (vision loss present) and 2 (vision loss and seizures present) for each of the cognitive tests. The proportion of participants able to recite the alphabet also decreased from Stage 1 to Stage 2 (*χ* ^2^ = 12.1, *p* < 0.01). Cognitive ability declines with advanced disease severity in CLN3 disease, though motor disability in Stage 3 likely contributes to difficulty participating in cognitive assessment at this later disease stage. Understanding the relationship between cognition and CLN3 disease stage may help guide decision making, that is, determining who could or should undergo cognitive assessment for clinical care or for group stratification in disease modifying clinical trials.

## Synopsis

Cognitive ability declines with advanced disease severity in CLN3 disease.

## 1 Introduction

The neuronal ceroid lipofuscinoses (NCLs; Batten disease) are inherited lysosomal storage disorders characterized by the accumulation of intracellular autofluorescent lipopigment. As a group, they are the most prevalent childhood neurodegenerative diseases [1]. CLN3 disease is an autosomal‐recessively inherited NCL caused by pathogenic variants in the *CLN3* gene [1].

In most affected individuals, CLN3 disease begins during childhood and is characterized by vision loss, cognitive decline, seizures, and motor decline; an uncommon ‘vision loss’ only phenotype has also been described [2, 3]. Among individuals with a classic presentation, progressive functional decline occurs from symptom onset until eventual death in the third decade of life [4, 5]. Vision loss is often the first reported symptom, with average age of onset between 4 and 7 years of age, followed by cognitive decline starting between 7 and 10 years of age, seizures beginning between 10 and 12 years of age, and emerging parkinsonism starting at age 11–13 years old [5, 6]. Behavioral problems also may develop between approximately 8 and 10 years of age [7].

The predictable manner of symptom change over time supported development of the CLN3 Staging System (CLN3SS). A higher CLN3SS stage reflects more advanced disease, and is associated with older chronological age, reflecting age‐related disease progression. The CLN3SS is based on the sequential onset of three cardinal disease events: vision loss, seizures, and loss of independent ambulation, but does not include another core feature, cognitive impairment [8]. Cognitive change in CLN3 disease is characterized by an initial attenuation or slowing in acquisition of new cognitive skills, followed by plateau and then decline, that is, loss of previously attained abilities [9]. However, it is unclear whether cognitive symptoms change in relation to other cardinal features (vision loss, seizures, loss of ambulation) of CLN3 disease. Because the CLN3SS provides well‐defined, objective inflection points for disease progression, we examined the association between the CLN3SS and cognitive changes. We hypothesized that cognitive test scores would be lower (i.e., worse performance) in association with higher CLN3SS stage (i.e., more advanced disease).

## 2 Methods

### 2.1 Participants

Eligible participants were individuals with a genetically confirmed diagnosis of CLN3 disease enrolled in the University of Rochester Batten Center (URBC) a prospective longitudinal natural history study. Study visits were conducted between July 2003 and June 2024 at the URBC, and at annual meetings of the Batten Disease Support and Research Association (BDSRA) Foundation using URBC mobile or remote research lab methods [10, 11]. For this investigation, we included individuals with at least one evaluation with the Unified Batten Disease Rating Scale (UBDRS) [12] from which CLN3SS stage could be derived, and neuropsychological evaluation within 6 months of the UBDRS evaluation. Genetic diagnosis was established through review of available records provided by participants' caregivers (usually parents) or research genetic testing completed at the URBC [13].

### 2.2 Measures

#### 2.2.1 Neuropsychological Assessment

For the present analysis, we focused on four subtests from our research battery, specifically the Wechsler Intelligence Scale for Children fourth and fifth editions (WISC‐IV; WISC‐V): Vocabulary, Information, Similarities, and Digit Span. These tasks assess, respectively, vocabulary knowledge, fund of information (e.g., general knowledge), verbal reasoning, auditory attention (Digit Span Forward) and working memory (Digit Span Backward). Subjects were also asked to recite the full alphabet independently; “singing” the alphabet song was acceptable. Most if not all affected individuals will have learned and mastered alphabet singing and recitation prior to symptom onset or diagnosis. Due to vision loss in affected individuals, all tasks were verbally administered and required only verbal responses. Full details of the battery have been previously described [14]. Except for correct, complete alphabet recitation (scored as 1 = pass; 0 = fail), analysis of cognitive test performance focused on age‐adjusted scaled scores (s.s.), which reflect performance in relation to same‐age peers. s.s. have an average = 10 (standard deviation, SD = 3); average scores range from s.s. = 6 (low average) to s.s. = 14 (high average).

The long timespan for data collection resulted in a version update from WISC‐IV to WISC‐V. WISC subtest scores are comparable across these two test versions for individuals with NCL disorders [15]. Therefore, data were combined in the present analyses; hereafter ‘WISC’ in the present paper references data from either test version.

#### 2.2.2 CLN3SS

The CLN3SS is a rank‐ordered scale (range: 0–3) capturing the presence of three cardinal disease‐related events elicited through the UBDRS exam and review of medical history. Each one‐point rank increase in CLN3SS reflects onset of one additional, cardinal event. CLN3SS Stage 0 = genetic diagnosis only; Stage 1 = genetic diagnosis + vision loss; Stage 2 = genetic diagnosis + vision loss + onset of one or more seizures; Stage 3 = genetic diagnosis + vision loss + seizure onset + cannot walk independently (Figure 1). CLN3SS stages previously assigned in the master dataset were re‐confirmed by review of all available data for a respective subject. Only subjects with a confirmed CLN3SS were included in the analysis.

**FIGURE 1:**
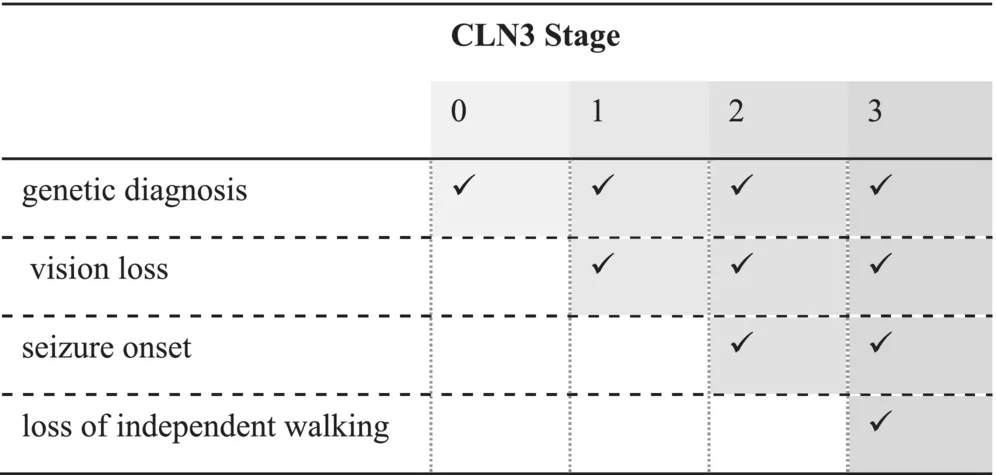
CLN3 Staging System (CLN3SS).

### 2.3 Data Analysis

Data were analyzed and graphs were produced using JMP Student Edition version 18.2 [16]. Repeated measures analysis of variance (ANOVA) tests were used to examine differences in WISC s.s by group, with group defined by CLN3SS. Post hoc Tukey tests were used for pairwise comparisons of cognitive test scores by CLN3SS stage. A chi‐square test was also used to evaluate the relationship between CLN3SS and the ability to recite the alphabet independently (yes/no). Data from each paired evaluation (neuropsychological evaluation + CLN3SS) for each subject were included in these analyses. For the Digit Span analysis, we examined the two individual components of the test that are shared across each WISC version (Digit Span Forward; Digit Span Backward).

## 3 Results

### 3.1 Sample Characteristics

Analyses were performed on 245 paired assessments (WISC + CLN3SS) from *n* = 84 unique individuals (47 males, 37 females), who completed between 1 and 14 visits (mean = 2.8 visits, SD = 2.7, median = 2, mode = 1). Fifty two participants were homozygous for the common 1‐kb deletion, and 23 were heterozygous for the common deletion and a non‐common mutation. One subject was homozygous for a non‐common pathogenic variant, and 8 had genetic confirmation without full details of their mutation type (e.g., mutation details on only one allele reported, typically this was the common variant). Table 1 shows the number of subjects by the number of paired assessments completed. Based on the most recent evaluation for each subject, the mean age was 13.7 years old (SD = 4.3, range = 6.4–27.1; data were reasonably normally distributed; skew = 0.39; kurtosis = 0.15). Vision loss was present in every subject at their first assessment (i.e., no CLN3SS = 0 participants) and the mean age for vision loss was 6.1 years old (SD = 1.5 years, range = 3.0–12.0 years). Twenty two individuals (26.2%) had never experienced seizures over the course of their study participation. For the *n* = 62 (73.8%) with a positive seizure history, age of onset was available for *n* = 60. Among those participants, the mean age of seizure onset was 9.6 years old (SD = 2.6, range = 0.8–15.3 years). At the first assessment completed for each participant, the number of individuals within each CLN3SS group was: CLN3SS = 1, *n* = 33; CLN3SS = 2, *n* = 49; CLN3SS = 3, *n* = 2. At the last assessment, the number within each CLN3SS group was: CLN3SS = 1, *n* = 21; CLN3SS = 2, *n* = 52; CLN3SS = 3, *n* = 11. The shift to more subjects in later CLN3SS stages is largely explained by disease progression in affected individuals with repeat assessments.

**TABLE 1:** Number of subjects by number of study visits completed.

Number of subjects	Number of visits completed
37	1
15	2
6	3
8	4
5	5
6	6
2	7
1	8
2	9
1	13
1	14

### 3.2 Cognitive Test Performance and CLN3DSS

At the first assessment, the mean score on each WISC subtest was within the below average range in comparison to age‐normative data. Table 2 presents all participants' WISC scores at the first assessment, across all CLN3SS groups. All WISC scores decreased with increasing age of the sample (Table 3) but bivariate correlations between each WISC score and age were non‐significant. This may be driven by floor effects of the WISC scores evident even at young ages. However, repeated measures ANOVA tests revealed a significant difference by CLN3SS score for each of the WISC tests analyzed: Similarities, *F* (2, 148.8) = 12.83, *p* < 0.0001; Vocabulary, *F* (2, 202.5) = 21.16, *p* < 0.0001; Information, *F* (2,147.3) = 17.32, *p* < 0.0001; Digit Span Forward, *F* (2, 217.8) = 18.53, *p* < 0.0001; Digit Span Backward, *F* (2, 149.3) = 6.34, *p* < 0.01. Figure 2 present boxplots to show the relationship between WISC subtest scores and disease stage as defined by CLN3SS. Post hoc testing (Tukey; results shown in Table 4) showed that mean scores on all WISC tests were lower (i.e., worse performance) when subjects were at more advanced disease stages as defined by the CLN3SS (Stage 2 and Stage 3), as compared to CLN3 Stage 1. In addition, mean s.s. on the Information subtest was significantly lower for individuals in CLN3SS = 3, as compared to CLN3SS = 2.

**TABLE 2:** WISC‐IV or WISC‐V subtest scaled scores for all participants, all CLN3SS groups at their first assessment (*n* = 84 subjects).

WISC subtest	Mean (SD), range
Similarities (*n* = 50)	5.8 (2.8) 1–13
Vocabulary (*n* = 68)	4.7 (3.2) 1–15
Information (*n* = 48)	5.2 (3.1) 1–14
Digit Span Forward (*n* = 79)	4.2 (2.4) 1–11
Digit Span Backward (*n* = 56)	5.8 (3.2) 1–14

**TABLE 3:** WISC subtest scores for all assessments by CLN3 Staging System (CLN3SS) group.^a^

WISC subtest	Mean (SD), range [*n*]		
	CLN3SS = 1	CLN3SS = 2	CLN3SS = 3
Similarities	6.9 (2.8)	5.2 (3.0)	3.0 (1.8)
	1–13 [59]	1–15 [108]	1–5 [6]
Vocabulary	6.0 (3.0)	3.9 (3.2)	1.0 (0)^b^
	1–13 [69]	1–15 [140]	[9]
Information	6.4 (3.2)	4.4 (3.7)	1.4 (0.5)
	1–14 [58]	1–15 [108]	1–2 [7]
Digit Span Forward	5.4 (2.4)	3.6 (2.3)	1.5 (0.9)
	1–11 [68]	1–10 [150]	1–4 [13]
Digit Span Backward	6.5 (3.5)	4.8 (3.2)	1.7 (1.2)
	1–15 (65)	1–13 [112]	1–3 [3]

^a^ CLN3SS = 1: vision loss only; CLN3SS = 2: vision loss + seizures; CLN3SS = 3: vision loss + seizures + loss of independent ambulation.

^b^ Range is unavailable: all subjects received a scaled score = 1, that is, the floor of the measure.

**FIGURE 2:**
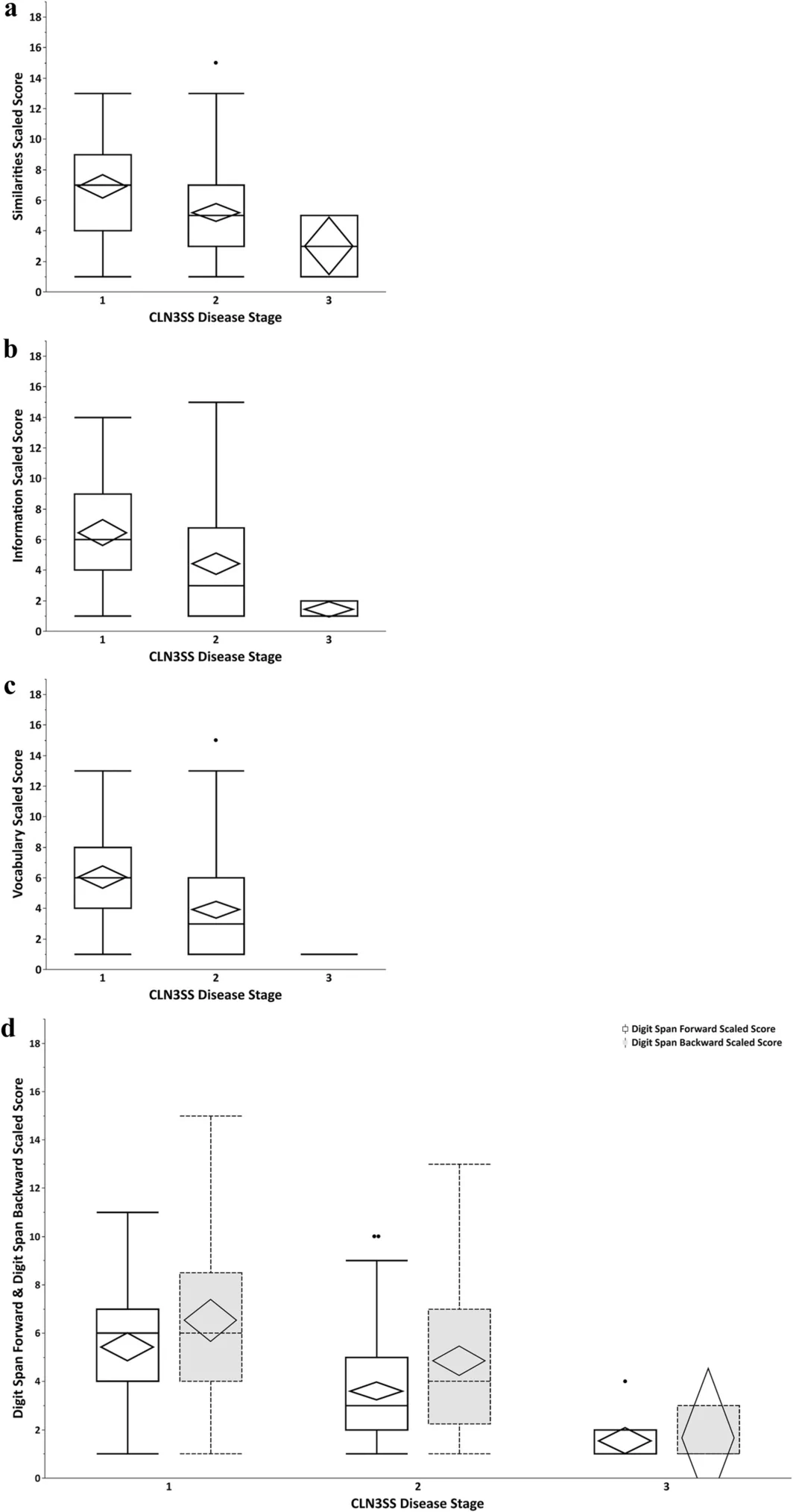
(a–d) Mean, 95% confidence intervals, and boxplots show the relationship between CLN3 Staging System (CLN3SS) disease stage and performance on Wechsler Intelligence Scale for Children (WISC) subtests. (a) Relationship between CLN3SS and WISC Similarities subtest. (b) Relationship between CLN3SS and WISC Information subtest. (c) Relationship between CLN3SS and WISC Vocabulary subtest. (d) Relationship between CLN3SS and WISC Digit Span Forward (DSF) and Digit Span Backward (DSB) subtest. Diamonds represent mean and 95% confidence intervals for WISC scaled score. Box and whisker plots present median, quartile, and range values for WISC scaled scores. Individual points show outlier values that are beyond 1.5 times the interquartile range.

**TABLE 4:** Post hoc (Tukey) comparisons for differences between CLN3SS groups on WISC subtest scores.

	Mean difference with group	*p*	95.00%
			Confidence interval
WISC Similarities			
CLN3 Stage 1	CLN3 Stage 2 = 1.9	**< 0.0001**	0.94, 2.9
	CLN3 Stage 3 = 3.1	**< 0.001**	1.2, 5.1
CLN3 Stage 2	CLN3 Stage 3 = 1.2	0.19	−0.5, 2.9
WISC Vocabulary			
CLN3 Stage 1	CLN3 Stage 2 = 2.4	**< 0.0001**	1.1, 3.2
	CLN3 Stage 3 = 3.9	**< 0.0001**	1.1, 2.4
CLN3 Stage 2	CLN3 Stage 3 = 1.6	0.08	−1.5, 3.3
WISC Information			
CLN3 Stage 1	CLN3 Stage 2 = 2.3	**< 0.0001**	1.2, 3.3
	CLN3 Stage 3 = 4.1	**< 0.0001**	2.2, 6.0
CLN3 Stage 2	CLN3 Stage 3 = 1.8	**< 0.05**	0.1, 3.5
WISC Digit Span Forward			
CLN3 Stage 1	CLN3 Stage 2 = 1.7	**< 0.0001**	1.0, 2.6
	CLN3 Stage 3 = 3.0	**< 0.0001**	1.6, 4.4
CLN3 Stage 2	CLN3 Stage 3 = 1.2	0.06	−0.04, 2.5
WISC Digit Span Backward			
CLN3 Stage 1	CLN3 Stage 2 = 1.4	**< 0.01**	0.3, 2.4
	CLN3 Stage 3 = 3.3	**< 0.05**	0.3, 6.2
CLN3 Stage 2	CLN3 Stage 3 = 1.9	0.3	−0.9, 4.7

*Note:* Bold values indicate significant *p* values.

We also examined participants' ability to recite the alphabet independently. Age at the first assessment was not associated alphabet accuracy: (*χ* ^2^ (1, *N* = 60) = 0.05, *p* > 0.81). However, there was an association between this skill and disease stage. Figure 3 presents a mosaic plot showing differences amongst CLN3SS disease stages (Stages 1–3) in the ability to accurately recite the alphabet. Of a total of 197 assessments in which alphabet recitation was evaluated, accurate alphabet recitation was seen in 46/64 (72%) assessments in CLN3 Stage 1, 62/125 (50%) in CLN3 Stage 2, and 2/8 (25%) in CLN3 Stage 3. A chi‐square test showed a significant association between CLN3 disease stage and ability to recite alphabet, *χ* ^2^ = 12.1, *p* < 0.01.

**FIGURE 3:**
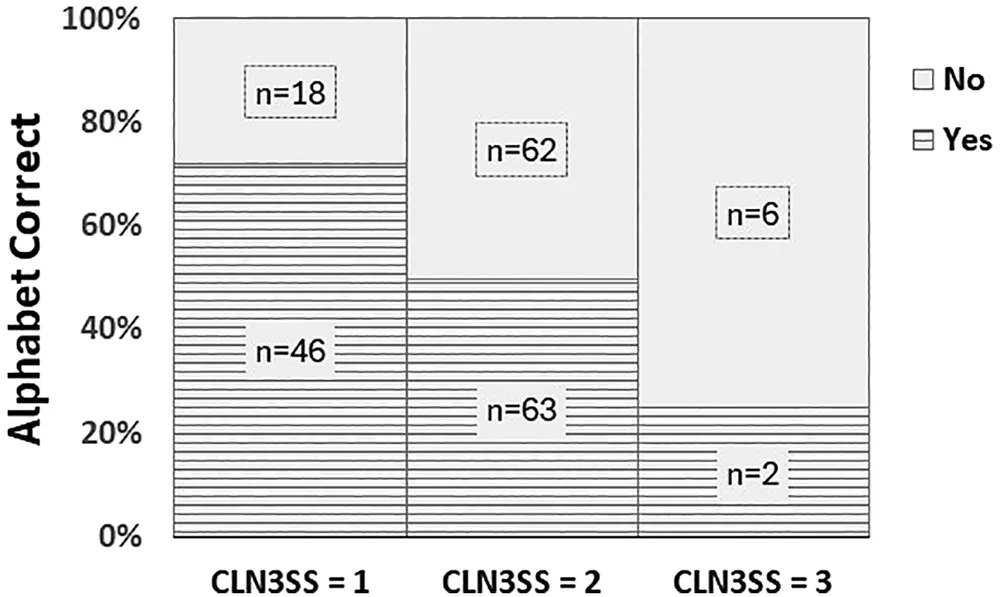
Mosaic plot of proportion and number of individuals able to recite alphabet correctly, by CLN3SS disease stage.

## 4 Discussion

The present study investigated the relationship between cognitive test performance and scores on the CLN3 Disease Staging System. We found that WISC scores on tests of vocabulary, verbal reasoning, general knowledge, attention, and working memory were significantly lower in CLN3SS disease Stages 2 and 3, compared to Stage 1, and that the score on a WISC test of general knowledge was significantly lower in CLN3SS disease Stage 2, compared to Stage 3. The ability to recite the alphabet also decreased with advancing disease severity. Most children learn the alphabet song during preschool years and by the end of their Kindergarten year are expected to know all letter names and their associated sounds [17]. As such, alphabet recitation may be considered as a ‘pre‐morbid’ skill, acquired before symptom onset and diagnosis for most individuals with CLN3 disease. The sharp decrease in the number of participants from Stage 2 to Stage 3 who could even attempt alphabet recitation also reflects disease burden, that is, dysarthria and eventual loss of speaking ability. Finally, even at CLN3SS Stage 1, participants' mean WISC scores were below average in comparison to same age peers, underscoring that even in an early stage of disease, cognitive function is impaired.

There are several study limitations. Participants' ability to complete study visits on an annual basis varied depending on ability to travel to the URBC or the annual family support meetings for study participation, or because they were present but unable to take part in cognitive assessment, for example, due to fatigue, recent seizure, or behavioral/psychiatric symptoms. For similar reasons, some participants completed some but not all tests within the cognitive battery, resulting in unequal numbers across the analyses of the relationship between the CLN3SS and each WISC subtest. Next, by using age‐adjusted scores (s.s.), floor effects were seen, meaning that some participants could participate in testing and correctly answer some items but attained the lowest possible age‐corrected score on the test. Floor effects can occur when a measure lacks sensitivity to detect differences at the low end of the ability scale [18]. We considered other metrics to evaluate cognitive change. Raw scores describe correct responses on a test without reference to age (e.g., the total sum of points earned), with higher scores typically indicating better performance, that is, more correct responses given. Though raw scores can minimize floor effects they have ordinal scale properties—a one point change in raw score does not reflect an equivalent change in ability across all points on the scale and limits comparability of scores. Growth scale value (GSV) scores also order performance by difficulty level, but apply item response theory/Rasch modeling to establish interval scaling so that the same amount of score change signifies the same amount of change in ability, across the entire span of a test [19, 20]. GSV scores are not available for Wechsler subtests, but are available for some measures of adaptive function. Our group has previously shown utility of Vineland Adaptive Behavior Scales‐third edition (Vineland‐3) GSV scores to illustrate change in adaptive function, though not yet with a specific focus on the CLN3SS; this work is in progress but is not the focus of the current project [21].

Finally, the majority of participants in this study were classified as CLN3SS = 1 or CLN3SS = 2. The lack of individuals in CLN3SS = 0 (genetic diagnosis only) is a broad limitation in all studies of CLN3 disease; few affected individuals receive a genetic diagnosis until after one or more symptoms emerges [5]. The diagnostic odyssey for CLN3 disease can be complicated by the rarity of this condition and because early symptoms are non‐pathognomonic; recently published diagnostic guidelines advise genetic screening following onset of vision loss [22]. This results in many individuals with a definitive genetic diagnosis of CLN3 disease assigned only after progression to CLN3SS = 1. On the other end of the CLN3SS spectrum, there were likely two contributors to the small sample size for individuals in CLN3SS = 3. First, assessments were conducted at the annual BDSRA meeting and the URBC, necessitating travel to the location of study visits. This created potential for selection bias, as travel may not be possible for participants in some years, and becomes more challenging over time due to increasing medical fragility and a higher level of care needs. Relatedly, the higher level of disability at CLN3SS = 3, particularly the loss of the ability to speak, or speak legibly, may have precluded participation in the cognitive assessment.

Results of the present study suggest that loss of the ability to participate in cognitive assessment may be a common associated feature among many affected individuals who have reached CLN3SS Stage 3. This may be helpful for stratifying individuals for assessment and clinical management, for example, consideration of whether those who have reached CLN3SS = 3 require cognitive assessment to guide clinical decision making (e.g., recommendations for school‐based accommodations), or whether resources can be better directed to inform other care needs. As well, an understanding that cognitive assessment may have limited applicability for many individuals at CLN3SS = 3 may guide trial design, that is, an intentional decision to not require cognitive assessment for individuals those at this disease stage who otherwise qualify for study participation. Finally, even amongst participants at CLN3SS = 3 able to complete cognitive assessment, the average age‐adjusted WISC subtest test scores were largely at or near the floor (s.s. = 1) of the measure, meaning that subjects earned the lowest possible score on the test. In the present study, the findings suggest that the metric of WISC s.s. may not be useful for sensitive estimation of change in a trial setting, particularly amongst individuals who can still complete assessment but may demonstrate floor effects on the test, and/or whose disease severity limits the ability to actively participate in performance‐based assessments of cognition. Future work is needed to understand whether there is a relationship between cognitive test performance and the CLNSS at Stage 0 (genetic diagnosis), to explore the relationship between adaptive function and CLN3 disease stage, and to more fully explore the characteristics of a larger sample of individuals in CLN3SS Stage 3 who retain adequate cognitive and speech abilities to support participation in cognitive assessment.

## Author Contributions

**Samuel D. Moran:** conceptualization, methodology, original draft preparation, review and editing. **Erika F. Augustine:** methodology, review and editing. **Jonathan W. Mink:** methodology, review and editing. **Marianna Pereira‐Freitas:** review and editing; **Naomi S. Taggart:** review and editing. **Jennifer Vermilion:** methodology, review and editing. **Amy E. Vierhile:** methodology, review and editing; **Heather R. Adams:** conceptualization, methodology, original draft preparation, review and editing.

## Funding

The following sponsors provided funding to support this research: University of Rochester Medical Education Awards Program; NIH grants U54NS065768, R01NS060022, K23NS058756, and U01NS101946; Batten Disease Support, Research, and Advocacy (BDSRA) Foundation; Luke and Rachel Batten Foundation; Noah's Hope‐Hope 4 Bridget Foundation; Our Promise to Nicholas Foundation; and Wilbur Smith Pediatric Neurology Fund at the University of Rochester.

## Ethics Statement

The research protocol was reviewed by the University of Rochester Research Subjects Review Board (IRB) and determined to meet federal and University criteria for exemption, based on the study nature, which was limited to secondary analysis of existing, de‐identified data (RSRB STUDY #9390).

## Conflicts of Interest

Heather R. Adams received grant support (payments to institution) from: NIH U54HD122210; consulting fees (payments to institution) from: Polaryx Therapeutics Inc. Erika F. Augustine received grant support (payments to institution) from: NIH U54HD122210, Beyond Batten Disease Foundation, and BDSRA Foundation; royalties from Up To Date; and consulting fees from Latus Bio. Jonathan W. Mink received consulting fees from Polaryx Therapeutics Inc. and Neurogene Inc. Jennifer Vermilion received grant support (payments to institution) from: NIH U54HD122210 and BDSRA Foundation Center of Excellence; consulting fees (payments to institution) from: Polaryx Therapeutics Inc.; support for attending meetings and/or travel from BDSRA Foundation; Dr. Vermilion is the Chair of the BDSRA Foundation Center of Excellence (unpaid position). Samuel D. Moran received grant support from the University of Rochester Medical Education Awards Program to conduct this research. The other authors declare no conflicts of interest.

## Data Availability

De‐identified data may be shared upon reasonable request and in compliance with institutional policy and a data sharing agreement.
